# The burden of infectious and cardiovascular diseases in India from 2004 to 2014

**DOI:** 10.4178/epih.e2016057

**Published:** 2016-12-14

**Authors:** Kajori Banerjee, Laxmi Kant Dwivedi

**Affiliations:** International Institute for Population Sciences, Mumbai, India

**Keywords:** Cardiovascular diseases, Hospitalization, Infectious disease, Share of in-patients death, Prevalence, Socioeconomic factors

## Abstract

**OBJECTIVES:**

In India, both communicable and non-communicable diseases have been argued to disproportionately affect certain socioeconomic strata of the population. Using the 60th (2004) and 71st (2014) rounds of the National Sample Survey, this study assessed the balance between infectious diseases and cardiovascular diseases (CVD) from 2004 to 2014, as well as changes in the disease burden in various socioeconomic and demographic subpopulations.

**METHODS:**

Prevalence rates, hospitalization rates, case fatality rates, and share of in-patients deaths were estimated to compare the disease burdens at these time points. Logistic regression and multivariate decomposition were used to evaluate changes in disease burden across various socio-demographic and socioeconomic groups.

**RESULTS:**

Evidence of stagnation in the infectious disease burden and rapid increase in the CVD burden was observed. Along with the drastic increase in case fatality rate, share of in-patients deaths became more skewed towards CVD from 2004 to 2014. Logistic regression analysis demonstrated a significant shift of the chance of succumbing to CVD from the privileged class, comprising non-Scheduled Castes and Tribes, more highly educated individuals, and households with higher monthly expenditures, towards the underprivileged population. Decomposition indicated that a change in the probability of suffering from CVD among the subcategories of age, social groups, educational status, and monthly household expenditures contributed to the increase in CVD prevalence more than compositional changes of the population from 2004 to 2014.

**CONCLUSIONS:**

This study provides evidence of the ongoing tendency of CVD to occur in older population segments, and also confirms the theory of diffusion, according to which an increased probability of suffering from CVD has trickled down the socioeconomic gradient.

## INTRODUCTION

The ongoing global health transition, encompassing both epidemiological transformations and related social changes, is characterized by a shift in disease burden from communicable to non-communicable diseases (NCD). However, differences between developed and developing countries in the health transition are stark. Developed countries have limited the burden of various communicable diseases by improving sanitary systems, hygiene and public health services. In contrast, in a developing country like India, basic hygiene requirements such as proper toilets are still a dream for many, with approximately 67% of rural households and 13% of urban households still defecating in the open (Census of India, 2011). Increases in air pollutants, water poverty, and the lack of safe drinking water in many parts of India have led to the aggravation of communicable disease incidence due to high population density [1-4]. In contrast, NCD, such as cardiovascular diseases (CVD), which are usually caused due to changes in lifestyle accompanying globalization and technical advancements, were previously more prevalent in developed countries, accounting for 50% of all deaths there. However, the incidence of CVD has gradually increased in developing nations where approximately 28% of deaths, mostly in the working-age population, are due to CVD [5,6]. Globally, the burden of CVD is predicted to peak, and India is predicted to lose 43.5 million disability-adjusted life years due to CVD by 2020 [7].

Studies have shown that demographic and socioeconomic status have a close relationship with disease. In developed countries where the standard of living is higher than in a developing country like India, the health complications and lifestyle diseases are presumed to be different. Morbidity and mortality due to infectious diseases such as diarrhea, worm infestations, malaria, respiratory diseases, tuberculosis are usually considered a major burden in middle-income and low-income countries [8,9]. Infectious diseases not only pose a greater threat to the poorer sections of the population, but also push individuals into poverty [8,10]. In contrast, NCD have a more complicated pattern of spread among various socioeconomic and demographic groups. The chances of succumbing to CVD is higher among individuals with a lower socioeconomic status in developed countries such as the US, whereas in India, the prevalence of CVD was found to be higher among the wealthier and better educated [11,12]. The wealthier inhabitants of India are exposed to Westernization leading to lifestyle diseases in addition to acculturation. Although NCD such as diabetes, hypertension, high blood sugar and heart disease are thought of as diseases of the rich, it is the poor who face the distress of being pushed into debt and consequent poverty when faced with the sudden onset of such diseases in India [13-15]. Since CVD is usually considered to be a lifestyle disease, studies have been conducted showing the prevalence of CVD to be correlated with improved education and wealth status [12].

The current epidemiological phase in India is frequently described as involving a “double burden.” The aim of the present study was to assess and compare changes in the burden of infectious diseases and CVD in India over the past decade. Moreover, efforts were made to evaluate whether the changes in the prevalence of infectious diseases, CVD, or both over the course of the study (2004-2014) were due to changes in the probability of having the conditions or due to changes in the composition of particular subpopulations. In light of the scarcity of studies precisely characterizing the current stage of the epidemiological transition in India, it is urgently necessary to understand the extent of changes in the prevalence rates of the major communicable diseases and NCD and the reasons for those changes to assist in effective resource allocation and the development of health programs and policies to improve public health.

## MATERIALS AND METHODS

Data were obtained from the nationally representative sample collected in the 60th (2004) and the 71st (2014) rounds of the National Sample Survey (NSS) of India, with 385,055 and 335,499 sampled individuals, respectively, including the deceased. The survey describes various background characteristics of individuals and lists the diseases each individual suffered from in the 15 days prior to the survey or had been admitted to a hospital for in the last 365 days (Appendix 1).

The burden of infectious diseases and CVD was calculated as follows:

(1) $$\frac{\mathrm{Number}\;\mathrm{of}\;\mathrm{persons}\;\mathrm{suffering}\;\mathrm{from}\;a\;\mathrm{selected}\;\mathrm{disease}\;\mathrm{at}\;a\;\mathrm{given}\;\mathrm{time}}{\mathrm{Total}\;\mathrm{number}\;\mathrm{of}\;\mathrm{people}\;\mathrm{surveyed}}\times 1,000$$

(2) $$\mathrm{Hospitalization}\;\mathrm{rate}=\frac{\mathrm{Number}\;\mathrm{of}\;\mathrm{persons}\;\mathrm{hospitalized}\;\mathrm{due}\;\mathrm{to}\;a\;\mathrm{selected}\;\mathrm{disease}\;\mathrm{at}\;a\;\mathrm{given}\;\mathrm{time}}{\mathrm{Total}\;\mathrm{number}\;\mathrm{of}\;\mathrm{people}\;\mathrm{surveyed}}\times 1,000$$

(3) $$\mathrm{Case}\;\mathrm{fatality}\;\mathrm{rate}=\frac{\mathrm{Number}\;\mathrm{of}\;\mathrm{deaths}\;\mathrm{from}\;a\;\mathrm{disease}}{\mathrm{Total}\;\mathrm{number}\;\mathrm{of}\;\mathrm{people}\;\mathrm{suffering}\;\mathrm{from}\;\mathrm{the}\;\mathrm{disease}}\times 1,000$$

(4) $$\mathrm{Share}\;\mathrm{of}\;\mathrm{in}-\mathrm{patients}\;\mathrm{death}\;=\frac{\mathrm{Number}\;\mathrm{of}\;\mathrm{in}-\mathrm{patients}\;\mathrm{deaths}\;\mathrm{caused}\;\mathrm{by}\;a\;\mathrm{paticular}\;\mathrm{disease}}{\mathrm{Total}\;\mathrm{number}\;\mathrm{of}\;\mathrm{deaths}}\times 1,000$$

The rate of change of prevalence from 2004 to 2014 was calculated using a simple relative change formula:

(5) Relative change in prevalence=∆P/PR(2004)

In this calculation, ΔP was calculated as PR(2014)−PR(2004), or the difference in disease prevalence in 2004 and 2014.

Prevalence changes over the 10-year period were analyzed using multivariate decomposition analysis. A logit model was used to estimate the effect of various socioeconomic and demographic variables on the presence of disease. The presence of disease was constructed as a dichotomous variable, with P=1 if a person suffered from a disease and P=0 otherwise. The statistical significance of the beta coefficients for these disease categories for the two time points was measured as:

(6) $$Z-\mathrm{test}=(b_{\left(2014\right)}-b_{\left(2004\right)})/\surd \mathrm{SE}(b_{\left(2014\right)}{)}^{2}+\mathrm{SE}(b^{\left(2004\right)}{)}^{2})$$

where b_(t)_=beta coefficients in the year t, (SE (b_(t)_) = standard errors of the beta coefficient in the year t, and t=2004, 2014.

Multivariate decomposition was used to decompose the difference in the prevalence in diseases over the study decade (2004-2014) into propensity (the change in the likelihood of suffering from the disease within various subgroups), composition (structural changes in the subgroups), and interaction effects (interplay of compositional changes and propensity of having the disease across various subgroups). Decomposition is a widely used measure to understand the reasons behind changes in the prevalence of a disease between two time points [16,17].

(7) $$\mathrm{logit}\;[\mathrm{NSS}\;71]-\mathrm{logit}\;[\mathrm{NSS}\;60]=(\beta_{0(\mathrm{II})}\;-\;\beta_{0(I)})\;+\;\sum \;P_{\mathrm{ij}(I)}\;(\beta_{\mathrm{ij}(\mathrm{II})}-\beta_{\mathrm{ij}(I)})+\;\sum \;\beta_{\mathrm{ij}(I)}\;(P_{\mathrm{ij}(\mathrm{II})}-P_{\mathrm{ij}(I)})\;+\;\sum (P_{\mathrm{ij}(\mathrm{II})}-P_{\mathrm{ij}(I)})*(\beta_{\mathrm{ij}(\mathrm{II})}-\beta_{\mathrm{ij}(I)})$$

where β_0_=regression constant, P_ij_=proportion of the j^th^ category of the i^th^ subgroup as calculated in Appendix 2, β_ij_=regression co-efficient of the j^th^ category of the i^th^ subgroup, and I and II stand for the 60th and 71st round of the NSS, respectively. In this equation, the 60th round of the NSS in 2004 was used as the base year.

### Ethics statement

No ethics statement is required, as this study was conducted using secondary data available in the public domain.

## RESULTS

### Overview of the disease burden

Although the prevalence of infectious diseases was higher than that of CVD, the prevalence of CVD almost doubled (from 7.34 to 13.48 per 1,000), whereas the prevalence of infectious diseases remained stagnant (from 29.57 to 28.05 per 1,000) over the study decade (2004-2014). The proportionate morbidity, which in this study was defined as the percentage of people suffering from a particular disease among all diseased people, for CVD in the 15 days immediately prior to the survey increased from 10% to 15%, but the proportionate morbidity of infectious diseases decreased from 2004 to 2014 (Table 1).

The prevalence rates of infectious diseases have decreased in many populous Empowered Action Group states, such as Rajasthan, Gujarat, Bihar, Madhya Pradesh, Maharashtra and Uttar Pradesh. A noticeable reduction was observed in the prevalence of infectious diseases in many northeastern states, such as Mizoram, Manipur, Meghalaya, Tripura, and Assam. The infectious disease prevalence was highest in Kerala for both of the study years, with no drastic change. For CVD, except for a minimal decline in Uttaranchal, Haryana, and Maharashtra, the prevalence increased in all states. The prevalence of CVD was not only found to be highest in Kerala, but also increased drastically from 32 to 82 people per 1,000 population in the study decade (Table 2).

**Figure 1.**
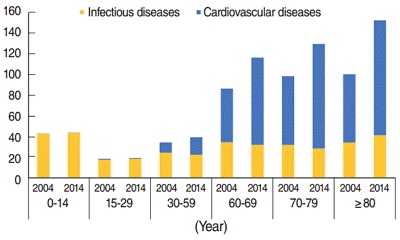
Prevalence of infectious and cardiovascular diseases from 2004 to 2014 by age.

The prevalence of CVD increased over the study decade for all subgroups of the population, whereas the prevalence of infectious diseases remained almost static. The prevalence of CVD increased significantly for all ages in 2014. However, the prevalence rate of infectious diseases was found to be greatest among younger age groups, with minimal differences over the course of the study (Figure 1). A marginal relative change in the prevalence of infectious disease was observed. However, the relative increase in the prevalence of CVD in 2014 was quite prominent for the rural population, Scheduled Castes/Tribes, the illiterate population with no formal education, and households with the lowest monthly expenditures (Table 3). Since the prevalence of CVD increased more sharply than that of infectious disease, this study attempted to identify which characteristics of the population this change could be attributed to.

The hospitalization rates for infectious diseases increased in many states, such as Uttar Pradesh, Jharkhand, Andhra Pradesh, Rajasthan, Sikkim, Assam, Meghalaya, Chhattisgarh, Himachal Pradesh, Haryana, Manipur, Karnataka, West Bengal, Madhya Pradesh, Punjab, Maharashtra, Tamil Nadu, Orissa, and Tripura. For the state of Kerala, although the hospitalization rate for infectious diseases declined from 2004 to 2014, it was still the highest in both years. The hospitalization rates for CVD were found to have risen in most states, including Kerala, where it was found to be the highest for both study years (Table 2).

**Figure 2.**
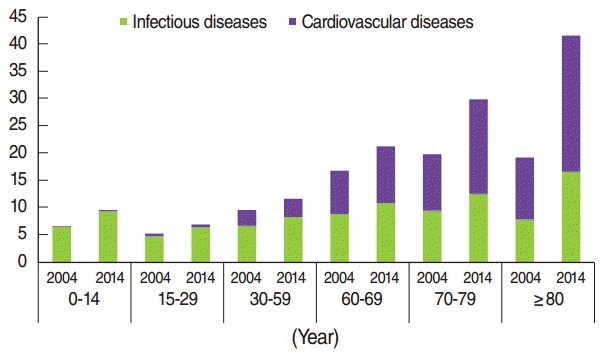
Hospitalization rates of infectious and cardiovascular diseases from 2004 to 2014 by age.

As the 60-plus population suffers more from CVD than younger age groups, the hospitalization rates are higher for the elderly. Therefore, a major concern may be the rise in hospitalization rate for CVD in the older members of the 30 to 59 year age group, whereas there is a noticeable rise in the prevalence of CVD in the earlier ages of the 30 to 59 subgroups (Figures 1 and 2).

The case fatality rate for inpatients who had been hospitalized at any time 365 days prior to the survey increased drastically for CVD, from 101 deaths per 1,000 population in the 60th round (2004) to 278 deaths per 1,000 population in the 71st round (2014). A smaller change was observed for infectious diseases. The share of in-patients deaths due to infectious diseases was slightly higher at 7.80% compared to CVD which stood at 6.64% in 2004. However, the share of in-patients deaths for infectious diseases increased to 10.77% whereas that for CVD doubled to 14.57% in 2014 (Table 1).

### Major factors influencing disease prevalence

In the previous section, a drastic rise in the prevalence of CVD was demonstrated, especially for those 30 years of age or older. The logistic regression model is run considering the 15 plus population as there is negligible prevalence of CVD in the ages below. The logistic regression model for this population estimated that females and urbanites had significantly greater odds of succumbing to CVD (1.16 at p<0.05 in 2004 and 1.15 at p<0.1 in 2014 for females; 1.67 at p<0.01 in 2004 and 1.52 at p<0.01 in 2014 for Urbanites). The likelihood of suffering from CVD showed a direct relationship with monthly household expenditures, while that of suffering from infectious diseases showed an inverse relationship. Significantly increased odds of suffering from CVD were found among the Other Backward Classes (1.49 in 2004 and 1.11 in 2014, at p<0.05 and p<0.01, respectively) and other social groups (1.80 in 2004 and 1.38 in 2014 at p< 0.01) compared to the Scheduled Castes/Tribes.

A decrease in the beta coefficients for many categories for both infectious diseases and CVD from 2004 to 2014 was noted. For example, the chance of having CVD among the Other Backward Classes was 0.40 (p<0.01) in 2004, which declined to 0.10 ( p<0.01) in 2014. Similarly, the chance of having CVD among the rich was 0.56 (p<0.01) in 2004, which declined to 0.36 (p<0.01) in 2014. The impact of education also significantly diminished, from 0.37 (p<0.01) in 2004 to 0.09 (p<0.01) in 2014. The Z-test showed that the decline in beta coefficients was not statistically significant for infectious diseases, indicating that the distribution of their burden remained similar for various subcategories from 2004 to 2014. However, for CVD, the decline in the beta coefficients was statistically significant according to social group, educational status, and monthly household expenditures. The reference categories for these characteristics were designed to include the underprivileged class of society in most cases, such as Scheduled Caste/Tribe populations, the illiterate and households with the lowest monthly expenditures. A statistically significant decline in beta coefficients suggests that the chance of having CVD increased among the reference categories. Thus, the logistic regression results demonstrate that the chance of having CVD has spread to include classes previously thought of as safe (Table 4).

### Multivariate decomposition of cardiovascular diseases

Decomposition was only performed for CVD, as the prevalence of infectious diseases did not change significantly. The change in the probability of having CVD explained 94.32% of the absolute change in its prevalence. Changes in the composition of the population and movement of subpopulations within and between strata contributed very little (only 0.29%) to explaining the change in the prevalence of CVD. The change in the rate of having CVD among individuals 15 years of age and older explained 12.43% of the absolute increase in the prevalence of CVD. This supports the theory of compression of morbidity, which states that due to improvement in life expectancies and medical science, the course of most diseases has been compressed into later stages of life. For many social categories, evidence of an increase in the propensity of having CVD among the groups that had a lower probability of having CVD was observed. Although a shift in the urban population took place, from 27% in 2004 to 32% in 2014 (Appendix 2), an increase in the rate of suffering from CVD was observed in rural areas, the reference category, when compositional changes were controlled for. Changes in the probability of having CVD in different social groups, educational categories, and monthly per-household expenditure quintiles explained 13.80, 5.90, and 9.51% of the increase in the prevalence of CVD, respectively. Changes in the composition of educational categories had the highest contribution among the compositional changes in increasing the prevalence of CVD. The rate of having CVD increased in the population with no formal education. The propensity of having CVD increased in the Scheduled Castes/Tribes, the never-married, households paying no medical insurance, and households with low monthly expenditures (Table 5).

## DISCUSSION

This study found sufficient evidence to support the proposal that India has entered the phase of the epidemiological transition characterized by stagnation in the pressure of infectious diseases but an increase in the burden of CVD. As predicted in previous researches, the share of deaths due to CVD overtook that of infectious diseases in the study decade [6,7]. The prevalence of infectious diseases was found to have decreased in many major states, such as Rajasthan, Gujarat, Bihar, Madhya Pradesh, Maharashtra, Uttar Pradesh, Mizoram, Manipur, Meghalaya, Tripura, and Assam, with an increase in the hospitalization rate in most of those states. This indicates the importance of prioritized targeting, effective medical measures, and health capacity enhancements. However, a major concern lies in the discrepancy of the prevalence and hospitalization rates of CVD among individuals 30 to 59 years of age (the working-age population), in which the prevalence has risen noticeably but hospitalization rate only marginally varied from 2004 to 2014.

Logistic regression confirmed a statistically significant increased chance of suffering from CVD among females, the elderly, urbanites, non-Scheduled Caste/Tribe categories, the more highly educated, households paying above Rs 1,000 annually for medical insurance premiums, and households with higher monthly expenditures. Notably, a significant decrease in the beta coefficients for the prevalence of CVD according to social group, educational status, and monthly household expenditures was observed, indicating an increase in the likelihood of suffering from CVD in the reference categories.

Multivariate decomposition found that the increase in the prevalence rate of CVD could be largely explained by an increase in the probability of having CVD in various population subgroups, rather than compositional shifts of the sampled population from 2004 to 2014. The rate of suffering due to CVD has increased among the rural population, contrary to previous beliefs that CVD is more prevalent among urbanites [18,19]. An increase in the propensity of suffering from CVD in Scheduled Castes/ Tribes, the poorly educated, and households with low monthly expenditures played a major role in the increase in the prevalence of CVD from 2004 to 2014. During the study period, a noteworthy compositional change occurred in education levels, with an overall increase observed in all educational categories, reflecting a general rise in enrollment and decrease in the dropout rate. This is highly significant, as improvements in education are directly linked with improvements in quality of life and hence changes in lifestyle, proving that NCDs are an unwanted byproduct of lifestyle changes.

Therefore, it is recommended to analyze changes in lifestyles and to implement effective public health measures. The burden of infectious diseases has remained more or less stagnant, necessitating immediate attention for its speedy reduction. However, judging by the findings of the present study, the term “double burden” may not be appropriate, as the burden is more skewed towards CVD. This study also brings to the forefront the fact that the burden of NCD has spread to the underprivileged classes of society previously thought to be safe. Poor households, households paying no medical insurance premiums, illiterate individuals, and Scheduled Castes/Tribes, which previously had a lower risk of CVD and a higher chance of having infectious diseases, are now burdened with both. This study provides evidence of the ongoing compression of CVD in the older ages of the population, and thereby confirms the theory of diffusion, according to which increased chances of suffering from CVD trickle down the social gradient.

Although the sampled population was limited, this study has imperative implications for the role of health care services and the objectives of the health sector in India. Policies impacting unplanned urbanization, the marketing of unhealthy food, and healthy-living initiatives need to be monitored to create a conducive environment for improved public health. An urgent need-assessment of the health resources and infrastructure available for the elderly to serve the older population suffering from CVD is required. Integrating NCD programs within existing health services and systems would probably be most effective. This study documented an increase in the burden of CVD among disadvantaged population groups, hinting at the importance of immediate control measures informed by analyzing the specific causes of this phenomenon and by transforming health insurance dynamics with a focus on the poorer sections of the population.

## Figures and Tables

**Table 1.** Comparison of the prevalence rates and mortality scenario of infectious and CVD between the 60th and 71st rounds of the NSS

Diseases	NSS 60th round, 2004						NSS 71st round, 2014					
	Weighted prevalence per 1,000 population	PM (%)	Total of persons suffering (n)	CFR per 1,000 population	SID (%)	Total of in-patients dead (n)	Weighted prevalence per 1,000 population	PM (%)	Total of persons suffering (n)	CFR per 1,000 population	SID (%)	Total of inpatients dead (n)
Infectious diseases	29.57	26.50	10,095	29.34	7.80	134	28.05	22.86	8,522	56.00	10.77	258
CVD	7.34	9.76	3,719	100.20	6.64	114	13.48	14.84	5,534	277.89	14.57	349
Subtotal of people suffering for both disease categories		36.26	13,814					37.70	14,056			
Total of affected people (n)			38,091						37,282			
Subtotal of in-patients dead due to both the disease categories					14.44	248					25.34	607
Total of dead people (n)						1,717						2,395

NSS, National Sample Survey; CVD, cardiovascular diseases; PM, proportionate morbidity; CFR, case fatality rate; SID, share of in-patients deaths

**Table 2.** Prevalence rates and hospitalization rates in various states of India in 2004 and 2014

States	Infectious diseases				Cardiovascular diseases				Sample size (n)	
	2004		2014		2004		2014			
	PR	HR	PR	HR	PR	HR	PR	HR	2004	2014
Jammu and Kashmir	20.84	2.76	11.38	6.31	2.79	1.16	12.54	3.43	6,777	6,826
Himachal Pradesh	19.07	5.55	19.56	10.55	9.26	2.12	10.53	3.19	7,151	4,436
Punjab	31.01	6.99	45.73	7.60	13.31	2.21	26.68	3.65	8,023	7,865
Chandigarh	16.85	2.78	53.16	7.62	15.34	2.41	16.03	1.75	1,805	879
Uttarakhand	23.58	2.19	38.19	3.58	6.22	1.18	2.06	1.52	2,646	3,185
Haryana	25.47	5.84	31.00	11.83	7.56	2.41	4.89	2.68	7,772	8,084
Delhi	3.20	2.66	20.26	7.72	1.91	2.41	1.78	2.58	5,206	5,445
Rajasthan	20.29	4.04	15.28	6.50	3.18	0.89	2.07	2.05	19,255	16,766
Uttar Pradesh	43.09	3.05	25.64	5.46	3.48	0.75	3.96	1.21	56,234	47,432
Bihar	26.72	2.74	23.58	2.72	1.59	0.45	3.10	1.78	23,851	17,719
Sikkim	13.50	4.23	15.02	5.68	0.70	1.18	5.10	1.05	2,547	2,112
Arunachal Pradesh	34.78	17.79	48.4	17.43	0.79	1.18	1.78	0.79	5,596	3,011
Nagaland	49.86	7.43	22.36	5.91	0.19	0.44	0.03	0.34	1,813	2,655
Manipur	15.09	5.85	9.90	9.61	1.29	1.91	1.21	0.80	8,609	7,212
Mizoram	9.20	11.93	8.10	9.33	0.31	0.47	0.41	1.23	5,374	3,892
Tripura	36.72	20.48	20.44	21.63	4.73	2.23	2.96	3.54	4,815	6,026
Meghalaya	30.00	4.83	14.47	7.75	0.79	0.30	0.04	1.13	4,057	4,401
Assam	44.08	4.45	15.48	4.21	4.28	0.30	0.39	1.00	14,091	11,494
West Bengal	28.44	6.10	45.37	8.23	11.96	1.51	17.78	2.83	24,904	22,999
Jharkhand	16.92	3.37	18.85	4.83	1.45	0.56	1.63	0.84	10,954	8,378
Orissa	35.16	8.57	43.33	12.45	1.63	0.69	8.42	1.60	13,003	11,657
Chhattisgarh	30.30	5.13	13.18	7.79	1.91	0.56	5.20	1.01	7,900	6,081
Madhya Pradesh	27.52	6.18	23.62	6.62	3.16	1.08	4.13	1.77	19,995	19,280
Gujarat	25.72	10.63	18.56	10.66	8.60	1.88	17.78	3.92	14,576	15,324
Daman and Diu	7.16	13.83	36.96	26.05	3.53	1.04	61.23	1.38	728	542
Dadra and Nagar Haveli	8.3 3	8.85	18.24	14.19	0.43	0.99	12.96	0.00	783	645
Maharashtra	28.24	8.13	27.15	10.20	11.02	2.70	8.27	2.80	26,578	27,292
Andhra Pradesh	21.09	3.86	23.33	12.24	13.35	2.03	44.70	5.21	22,387	10,731
K arnataka	17.97	5.92	27.64	11.13	6.29	1.47	17.79	3.26	16,986	14,834
G oa	21.87	9.06	39.51	4.93	8.32	2.97	32.09	3.46	903	922
Lakshadweep	29.87	14.22	21.59	7.55	16.03	6.18	52.80	10.71	962	842
K erala	52.24	26.92	52.63	24.97	31.88	9.47	81.19	11.82	13,333	11,319
Tamil Nadu	27.16	8.19	34.94	12.89	8.83	3.10	29.75	5.20	21,294	16,221
Pondicheri	14.16	7.63	47.24	5.03	29.10	5.49	52.90	5.01	1,185	1,122
Andaman and Nicobar Islands	22.71	16.02	11.93	11.60	5.65	3.69	37.11	4.42	1,245	1,245

PR, prevalence rate; HR, hospitalization rate.

**Table 3.** Prevalence of infectious and cardiovascular diseases by background characteristics

Background variables	Prevalence of infectious diseases per 1,000 population		Relative change of prevalence of infectious diseases	Prevalence of cardiovascular diseases per 1,000 population		Relative change of prevalence of cardiovascular diseases	Sample size (n)	
	2004	2014		2004	2014		2004	2014
Place of residence								
Rural	30.93	28.70	-0.07	4.86	9.65	0.99	251,984	190,904
Urban	25.59	26.51	0.04	14.63	22.44	0.53	133,079	144,595
Social group								
SC/ST	30.63	29.13	-0.05	3.15	7.84	1.49	110,271	98,596
OBC	30.52	27.92	-0.09	6.05	13.68	1.26	144,695	133,565
Others	27.75	27.45	-0.01	12.84	19.02	0.48	128,236	100,943
Education level								
Not literate/no formal schooling	36.52	38.30	0.05	5.43	14.08	1.59	209,710	106,505
Primary/middle	21.31	26.50	0.24	8.08	12.85	0.59	109,475	136,285
Secondary/higher secondary	17.39	18.42	0.06	12.37	12.77	0.03	44,744	64,268
Higher education	17.20	14.75	-0.14	18.66	17.28	-0.07	19,066	26,039
Monthly household expenditures								
Poor	31.22	31.83	0.02	3.91	9.89	1.53	138,999	117,057
Middle	30.17	25.34	-0.16	6.83	13.21	0.93	117,864	117,211
Rich	26.03	24.61	-0.05	14.80	21.95	0.48	126,449	98,800

SC/ST, Scheduled Caste/Scheduled Tribe; OBC, Other Backward Classes.

**Table 4.** Results of logistic regression for prevalence of infectious diseases and cardiovascular diseases in India, 2004 and 2014

Background variables	Infectious diseases^1^					Cardiovascular diseases^2^				
	NSS 60th round, 2004		NSS 71st round, 2014		Difference in β i2-i1	NSS 60th round, 2004		NSS 71st round, 2014		Difference in β c2-c1
	OR	β (i1)	OR	β (i2)		OR	β (c1)	OR	β (c2)	
Constant	0.02	-3.82	0.01	-4.35	-0.53		-8.44		-8.43	0.01
Sex (ref: male)										
Female	1.06	0.06	1.17^**^	0.16^**^	0.10	1.16^**^	0.15^**^	1.15^*^	0.14^*^	-0.01
Age (ref: 15-29 yr)										
30-59	1.51^**^	0.41^**^	1.16	0.15^**^	-0.26	8.76^**^	2.17^**^	12.81^**^	2.55^**^	0.38
60-69	1.63^**^	0.49^**^	1.38^**^	0.32^**^	-0.17	47.47^**^	3.86^**^	51.94^**^	3.95^**^	0.09
70-79	1.53^**^	0.43^*^	1.32^**^	0.28^**^	-0.15	68.72^**^	4.23^**^	65.37^**^	4.18^**^	-0.05
≥80	1.31	0.27	1.37^*^	0.32^*^	0.05	70.81^**^	4.26^**^	60.95^**^	4.11^**^	-0.15
Place of residence (ref: rural)										
Urban	1.01	0.01	1.04	0.04	0.03	1.67^**^	0.51^**^	1.52^**^	0.42^**^	-0.09
Social groups (ref: SC/ST)										
OBC	0.92	-0.08	0.98	-0.02	0.06	1.49^*^	0.40^*^	1.11^**^	0.10^**^	-0.30^†^
Others	0.73^**^	-0.32^**^	1.02	0.02	0.34	1.80^**^	0.59^**^	1.38^**^	0.32^**^	-0.27^†^
Education level (ref: not literate/ no formal education)										
Primary/middle	0.92	-0.08	0.95	-0.06	0.02	1.49^**^	0.40^**^	1.35^**^	0.30^**^	-0.10^†^
Secondary/higher secondary	0.87	-0.14	0.84	-0.17	-0.03	1.45^†^	0.37^†^	1.09^**^	0.09^**^	-0.28^†^
Higher education	0.77	-0.26	0.73	-0.31	-0.05	1.15^†^	0.14^†^	0.89	-0.12	-0.26^†^
Annual medical insurance premium paid by the household (ref: no premium paid)										
Below Rs1,000	1.71^*^	0.54^*^	1.16^†^	0.15ᅮ	-0.39	1.22^*^	0.20^*^	1.25	0.22	0.02
Above Rs1,000	1.26	0.23	0.92	-0.08	-0.31	1.95^**^	0.67^**^	1.43^**^	0.36^**^	-0.31
Monthly household expenditure (ref: poor)										
Middle	0.78^**^	-0.24^**^	0.86^**^	-0.15^**^	0.09	1.52^*^	0.42^*^	1.11^**^	0.10^**^	-0.32^†^
Rich	0.71^**^	-0.34^**^	0.76^**^	-0.27^**^	0.07	1.75^**^	0.56^**^	1.43^**^	0.36^**^	-0.20^†^

NSS, National Sample Survey; OR, odds ratio; β: beta coefficients of the regression; SC/ST, Scheduled Caste/Scheduled Tribe; OBC, Other Backward Classes; Rs, Indian rupee.

1 Models has been controlled for religion, marital status, type of latrine, type of drainage, type of source of energy, source of water and states.

2 Models has been controlled for religion, marital status and states.

† p<0.1

* p<0.05

** p<0.01

**Table 5.** Decomposition of changes in prevalence of cardiovascular diseases in India, 2004-2014 (by logit model)

Explanatory variable (Col 1)	Change			Absolute change	
	Rate (Col 2)	Composition (Col 3)	Interaction (Col 4)	Rate (Col 4)	Composition (Col 5)
Intercept	0.94				
Sex (ref: male)	-1.15	-0.14	0.00	0.24	0.03
Age (ref: 15-29 yr)	58.52	2.92	2.22	12.43	0.62
Place of residence (ref: rural)	-7.69	3.52	-1.15	1.63	0.75
Social group (ref: SC/ST)	-64.99	0.49	0.10	13.80	0.11
Religion (ref: Hindu)	-10.58	0.58	-1.04	2.25	0.12
Education level (ref: not literate no formal education)	-27.79	10.38	-12.81	5.90	2.21
Marital status (ref: never married)	-20.07	-2.75	0.01	4.26	0.58
Annual medical insurance premium paid by the household (ref: no premium paid)	-0.51	0.12	-3.23	0.11	0.02
Monthly household expenditures (ref: poor)	-44.76	1.66	-2.54	9.51	0.35
State (ref: Jammu Kashmir, Haryana, and Uttaranchal)	233.81	-0.71	-13.36	49.66	0.15
Total	115.74	16.06	-31.80		
Absolute change (%)	94.32	0.29	5.39		

Col, Column; SC/ST, Scheduled Caste/Scheduled Tribe.

## Appendix 1.

Definitions of the disease categories

## Appendix 2.

Proportions of people according to background characteristics
