# Supplementary material for: The burden of infectious and cardiovascular diseases in India from 2004 to 2014
Source: Epidemiol Health. 2016 Dec 14;38:e2016057. doi: 10.4178/epih.e2016057 (PMC5309727; doi:10.4178/epih.e2016057)
Supplement: Supplementary file 1 [file epih-38-e2016057-app.pdf]

Appendix 1. Definitions of the disease categories

Diseases	Definition	
	71st round, NSS (2014)	60th round, NSS (2004)
Infectious Diseases	Fever with loss of consciousness or altered consciousness, fever with rash/ eruptive lesions, fever due to diptheria, whooping cough, all other fevers (including malaria, typhoid and fevers of unknown origin, all specific fevers that do not have a confirmed diagnosis), filariasis, tetanus, tuberculosis	Malaria, eruptive mumps, diptheria, whooping cough, fever of unknown origin, tetanus, filariasis/ elephantiasis, tuberculosis
Cardiovascular diseases	Hypertension, heart diseases	Heart disease, hypertension

NSS, National Sample Survey.

Appendix 2. Proportions of people according to background characteristics

Background variables	P (2004)	P (2014)	P (2014) - P (2004)
Sex			
Male	0.51	0.51	0.00
Female	0.49	0.49	0.00
Age (yr)			
15-29	0.40	0.38	-0.02
30-59	0.49	0.51	0.02
60-69	0.07	0.07	0.00
70-79	0.03	0.03	0.00
≥80	0.01	0.01	0.00
Place of residence			
Rural	0.73	0.68	-0.04
Urban	0.27	0.32	0.04
Social groups			
SC/ST	0.27	0.27	0.00
OBC	0.40	0.44	0.04
Others	0.33	0.29	-0.04
Religion			
Hindu	0.83	0.82	-0.02
Muslim	0.11	0.13	0.02
Christianity	0.05	0.05	0.00
Education level			
Not literate/no formal schooling	0.48	0.30	-0.18
Primary/ middle	0.31	0.36	0.05
Secondary/higher secondary	0.15	0.25	0.10
Higher education	0.06	0.09	0.03
Marital status			
Never married	0.22	0.24	0.02
Currently married	0.70	0.68	-0.01
Divorced/widowed/separated	0.08	0.08	-0.01
Annual medical insurance premium paid by the household (Indian rupee)			
No premium paid	0.98	0.93	-0.05
<1,000	0.01	0.03	0.02
≥1,000	0.01	0.04	0.04
Monthly household expenditure			
Poor	0.46	0.45	-0.01
Middle	0.29	0.34	0.05
Rich	0.25	0.22	-0.03

P, proportion in ith year; SC/ST, Scheduled Caste/Scheduled Tribe; OBC, Other Backward Classes.
